# Green UPLC method for estimation of ciprofloxacin, diclofenac sodium, and ibuprofen with application to pharmacokinetic study of human samples

**DOI:** 10.1038/s41598-023-44846-5

**Published:** 2023-10-17

**Authors:** Alaa A. Ahmed-Anwar, Mahmoud A. Mohamed, Ahmed A. Farghali, Rehab Mahmoud, Mohamed E. M. Hassouna

**Affiliations:** 1 Chemistry Department, Faculty of Science, Beni-Suef University, Beni-Suef, 62514 Egypt; 2 Hikma Pharmaceutical Company, Beni-Suef, Egypt; 3 Materials Science and Nanotechnology Department, Faculty of Postgraduate Studies for Advanced Sciences, Beni-Suef University, Beni-Suef, Egypt; 4 Central Research Laboratory, Analytical Chemistry Department, Nahda University, Beni-Suef, Egypt

**Keywords:** Environmental sciences, Health care, Chemistry

## Abstract

Investigation of a unique and fast method for the determination and separation of a mixture of three drugs viz*.,* ciprofloxacin (CIP), Ibuprofen (IBU), and diclofenac sodium (DIC) in actual samples of human plasma. Also, the technique was used to look at their pharmacokinetics study. Hydrocortisone was chosen as the internal standard (IS). The drugs were chromatographically separated using an Acquity ultra-performance liquid chromatography UPLC ® BEH C18 1.7 µm (2.1 × 150 mm) column with a mobile phase composed of acetonitrile: water (65:35, v/v) adjusted to pH 3 with diluted acetic acid. Plasma proteins were precipitated with acetonitrile. The separated drugs ranged from 0.3 to 10, 0.2–11, and 1–25 µg/mL for CIP, IBU, and DIC, respectively. Calibration curves were discovered to achieve linearity with acceptable correlation coefficients (0.99%). Examination of quality assurance samples showed exceptional precision and accuracy. Following the successful application of this improved technique to plasma samples, the pharmacokinetic characteristics of each selected drug were evaluated using (UPLC) with UV detection at 210 nm. Two green metrics were applied, the Analytical Eco-scale and the Analytical GREEnness Calculator (AGREE). Separation was achieved in only 4-min analysis time. The method's validation agreed with the requirements of the FDA, and the results were sufficient.

## Introduction

**Figure 1:**
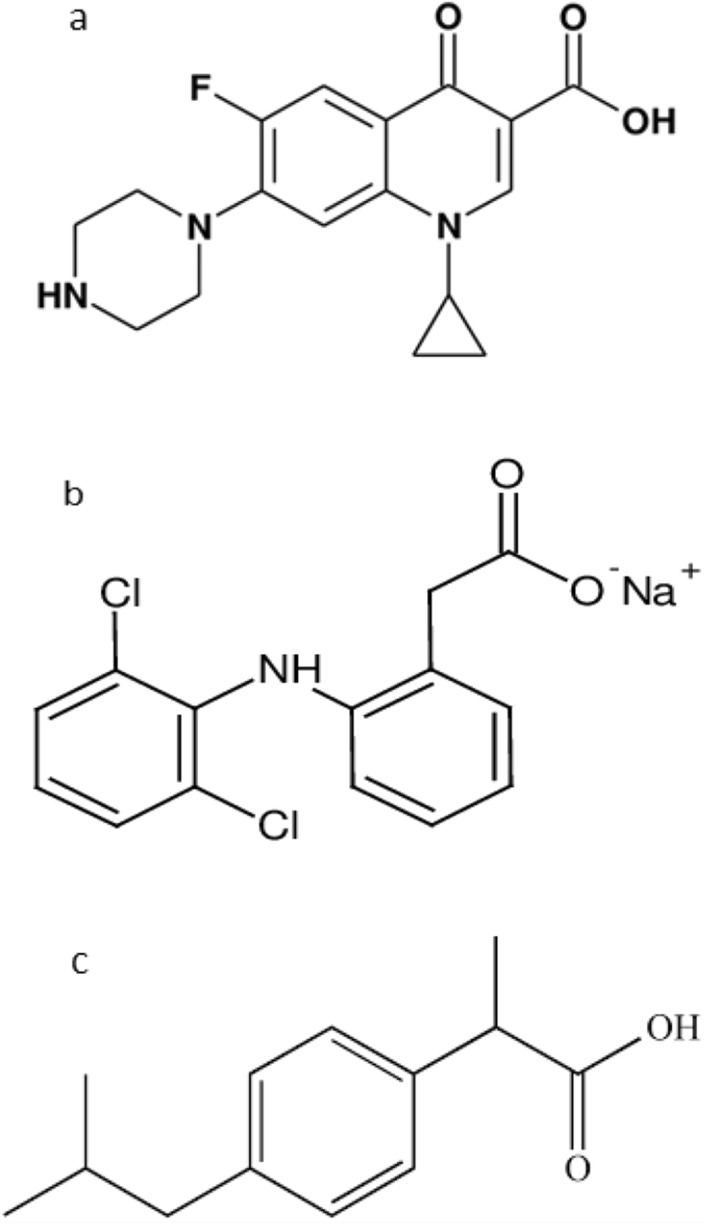
Structures of (**a**) CIP, (**b**) DIC, and (**c**) IBU.

CIP (1-cyclopropyl-6-fluoro-4-oxo-7-piperazin-1-ylquinoline-3-carboxylic acid) (Fig. 1a) is a fluoroquinolone antibiotic that is of the second generation and is widely used to overcome respiratory diseases and urinary tracts in mild to severe severity^1,2^.

DIC (2-[2-(2,6-dichloroanilino) phenyl] acetic acid) (Fig. 1b) is the salt of sodium version of DIC, a non-steroid anti-inflammatory medication (NSAID) has analgesic, antipyretic, and anti-inflammatory properties and is produced from benzene acetic acid. Diclofenac sodium decreases cyclooxygenase (COX) in a non-selective, reversible, and competitive manner, blocking the formation of prostaglandin precursors from arachidonic acid. As a result, prostaglandin production related to pain, inflammation, and fever is inhibited^3^.

IBU [2-(4-isobutyl phenyl) propionic acid], naproxen [6-methoxy-α-methyl-2-naphthalene acetic acid,] and tolmetin [1-methyl-5-(4-methyl benzoyl)-1*H*-pyrrole-2-acetic acid] Fig. 1c are non-steroid anti-inflammatory medicines (NSAIDs) that show favorable anti-inflammatory and analgesic characteristics^4^.

UPLC is a technique intended to improve sensitivity and accuracy while reducing solvent usage and processing time^5^. The detection of CIP in biological fluids has been the subject of numerous HPLC methods documented in the literature^6–10^. Liang et al., 2002 published a paper using reversed-phase HPLC with fluorescence and UV detection to separate and determine CIP and another five drugs in human plasma^11^. Few HPLC procedures were found to determine CIP, DIC, and IBU simultaneously. However, the number of techniques for separation is registered, and various medications for pain relief, inflammation, and infections are determined^12^. Green analytical chemistry aims to make it less dangerous, eco-friendly, and more economical for analysts. The bases of analytical chemistry greenness include reducing solvents, using ecologically friendly and low-toxic chemicals, decreasing waste output, analysis duration, and energy usage, and increasing integration, breakdown, and ease of use of analytical tools and procedures^13^.

The parts of chemical steps that follow the green chemistry principles are described in green chemistry metrics. Metrics are used to evaluate chemical processes' efficiency or environmental performance and measure performance improvements. In recent years, several green metrics have been created to assess how well analytical methods correspond to the green analytical chemistry principles. The national environmental metric index (NEMI) is one example of a qualitative tool^14^, analytical Eco-scale and green analytical procedure index GAPI^15,16^ are semi-quantitative, and AGREE (Analytical GREEnness Metric Approach and Software)^17^ is quantitative.

Separation of the drugs is carried out using UPLC. This study aims to create a unique, fast separation and determination procedure for CIP, IBU, and DIC in pharmaceutical formulations. The investigation' aim was increased to look at the three drugs' pharmacokinetic characteristics. The method's validation agreed with the requirements of the FDA, and the results were sufficient. According to all outcomes, the novel approach is the best in terms of making fixed dose combination available for the three drugs together, how it affects the environment and people's life and how much it costs to analyze data and prepare samples.

## Materials and methods

### Instruments

Dionex Ultimate 3000 UHPLC. It had an autosampler, a diode array detector, and a tetra solvent delivery pump (Massachusetts, USA). An X-Bridge Acquity UPLC ® BEH C18 1.7µm (2.1 × 150 mm) column was used for separation and quantification. The mobile phase and samples were filtered using 0.22 µm MS disposable syringe filters and 0.22 µm Millipore nylon membrane filters. The mobile phase was a mix of acetonitrile and water (pH 3 by diluted acetic acid). The separation system was 50% acetonitrile − 50% H_2_O, with a 0.2 mL/min flow rate. During the experiment, a Jenway pH meter was for adjusting the pH of the mobile phase (model 3510, Staffordshire, UK). Sonix TV SS-series ultrasonicator (South Carolina, USA) and a digital balance from Sartorius (Germany) were employed. The mixture was detected at 210 nm. The injection volume was 5 µL and analyzed for 4 min. Also, the heat of the column was 25 °C. A rongtai micropipette with a volume range of 0.1–500.0 µL (Mainland, Shanghai, China). 4000 rpm variable speed centrifuge (Zjmzym, China) with 8 × 20 mL capacity.

### Drugs and reagents

Pure samples of the drugs DIC, IBU, and CIB were kindly offered by native companies, viz*.,* Mina-pharm (10th of Ramadan, Egypt), Abbott Healthcare for the Pharmaceutical Industries (90Th Street, New Cairo, Cairo), and ORGANOPHARM for pharmaceutical and chemical industries (Obour City, Egypt), respectively. EPICO for Pharmaceutical Industries kindly offered a pure hydrocortisone sample.

Diclac 75 mg tablets, produced by SANDOZI (Mina-pharm company, Egypt, under license from Hexal AG, Germany). CIP 750 mg tablets made by ORGANOPHARM for pharmaceutical and chemical industries (Obour city, Egypt) and Brufen 600 mg tablets produced by Abbott Healthcare for the Pharmaceutical Industries (90th Street, New Cairo, Cairo), were purchased from the local market. Sigma Aldrich provided HPLC-grade acetonitrile and acetic acid. Plasma samples from the volunteers were obtained to create the calibration curves before the suggested drugs were administered (Approval No: NUB-034-023 by The Committee of Nahda University, Beni-Suef, Faculty of Pharmacy has approved the study protocol from the ethical point of view).

### Solutions and calibration samples

From the 1000 µg/mL stock solutions of each CIP, DIC, IBU, and hydrocortisone (IS), 2.5 mL were transferred to 25-mL volumetric flasks. Each is completed to volume with a diluent solution of acetonitrile and H_2_O (pH 3 by diluted acetic acid) (50/50, v/v) to obtain 100 µg/mL of each.

To construct the calibration curves for each drug, three sets of 10-mL measuring flasks were provided with aliquots of the later working solutions of each of CIP, IBU, and DIC covering the concentrations ranging from 0.3 to 10, 0.2–11, and 1–25 µg/mL, respectively, using the produced working standard solutions (100 µg/mL) and addition of 0.5 mL of the working standard solution of hydrocortisone (IS) to all the samples. Then, a solution composed of a 50/50 v/v mixture of acetonitrile and water was used to complete all flasks to the mark.

Standards for calibration were prepared in-vitro by transferring known concentrations of the working solutions of drugs of interest into three test tubes. Each sample was prepared by adding 200 µL of standard plasma and 50 µL of the working solution of the internal standard and mixing them on a vortex for 1 min. The separation of the plasma protein precipitate required an additional 10 min of centrifugation at 4000 rpm with the addition of 2 mL of acetonitrile. Samples having the concentration range 0.3–10, 0.2–11, and 1–25 µg/mL of CIP, IBU, and DIC, respectively, were obtained by putting them in 200 µL of a mixture of acetonitrile and H_2_O (pH 3 with diluted acetic acid) (50:50, v/v) after evaporating the solvent in a water bath at 80 °C. Quality control samples at low, moderate, and high values were generated using the same procedure. Concentrations of CIP (1, 4, and 10 µg/mL), DIC (3, 9, and 25 µg/mL), and IBU (0.5, 2.5, and 11 µg/mL), respectively, were put in direct refrigeration at twenty degrees Celsius till testing.

### Creating calibration curves and validating a procedure

Pair calibration curves were prepared, one for pure standards and the other for spiked plasma samples, Table 1, and were plotted for each drug. The prepared samples were evaluated in triplicates according to the approved method and under the working conditions of the HPLC instrument. Peak areas for both the internal standard and the analytes were noted. Regression equations were derived by graphing the resulting peak area ratio (the analyte's peak area divided by that of the internal standard's one) vs. the constant concentration. The suggested method also examined the prepared quality control tests, and concentrations were mathematically calculated using the regression equations for calibration curves of spiked plasma. The results have verified the proposed process by adhering to the rules for industry-bioanalytical method validation set forth by the US FDA^18^.

**Table 1:** The number and nominal concentrations of calibration points of Ciprofloxacin, Diclofenac sodium, and Ibuprofen.

CIP				DIC				IBU			
Pure samples		Plasma		Pure samples		Plasma		Pure samples		Plasma	
Conc (µg/mL)	Peak area	Conc (µg/mL)	Peak area	Conc (µg/mL)	Peak area	Conc (µg/mL)	Peak area	Conc (µg/mL)	Peak area	Conc (µg/mL)	Peak area
0.5	0.53	0.3	1.31	3	3.949	1	4.40	3	1.0457	0.2	0.87
0.75	0.97	0.5	2.19	10	9.001	1.5	6.53	5	1.99	0.5	2.17
1.5	2.26	1	4.39	19	18.608	2	8.76	10	4.318	0.8	3.60
2.5	3.84	1.5	6.67	30	29.63	3	13.09	15	6.641	1.5	6.71
6	9.88	2.5	11.00	50	49.904	5	22.03	20	9.01	2	8.99
7.5	12.28	3.5	15.67	80	77.717	7	30.58	30	13.343	2.5	10.90
13	21.66	4	17.66	100	99.953	8	35.98	40	17.752	3.5	15.29
15	25.20	5.5	24.16			9	39.58	50	22.38	4.5	19.69
		7.5	33.37			13	57.62	70	31.11	5.5	23.99
		8	35.74			20	89.79	80	35.85	8.5	38.35
		10	44.79			25	110.48	100	46.25	11	48.83

### Greenness profile determination

Various factors must be considered when determining how environmentally friendly an analytical methodology is, including the quantity and toxicity of used chemicals, the produced waste, the used power, the count of the steps in the process, miniaturization, and automation. Two green metrics were applied, the Analytical Eco-scale and the Analytical GREEnness Calculator (AGREE). The Eco-scale score is the initially stated method^19,20^. The total penalty points (depending on toxicity, reagents, power, and waste) were subtracted from 100. The researcher can measure whether the method is ideally green, acceptable, or not. Another metric is AGREE. A green tool can be considered a rapid quantitative technique that provides a score indicating how closely a technique agrees with the 12 essential rules of green analytical chemistry. It is derived from readily available and accessible software^21^.

### The pharmacokinetic study, medication administration, and extraction protocol

Ethical Approval letter number (NUB-034-023). Approval valid from 15 to 04-2023.

Samples of the target analytes were taken from the plasma of healthy, smoker-free individuals (some of the authors themselves) with an age range of 35–40 years and a weight range of 70–85 kg who had fasted for 8 h except for water without food and were measured using this method after the entire validation process was completed. The study’s potential participants have confessed their complete awareness with information about the goals and risks of the volunteering methodology before being requested to sign a written consent form which was further approved by the institutional ethics committee. ”An informed consent was obtained from all subjects/participants”.

For an oral dose of CIP (750 mg), DIC (75 mg), and IBU (600 mg), 3 groups are formed (n = 3). During the pharmacokinetic analysis, 3.5 mL of blood was drawn from the authors at times of 0.25–9 h. Ethylene diamine tetra acetic acid (EDTA) was used as an anticoagulant during the initial centrifugation of the blood samples at 4000 rpm for 10 min. The collected plasma samples were kept in a fridge until the day of the test. The suggested drugs were extracted from the samples by employing acetonitrile as a protein precipitant. A volume of 50 µL of the internal working standard's prepared solution (100 µg/mL) was added to 200 µL of each sample after placing it in a centrifuge tube. A plasma protein precipitant (2 mL) was added after a 1-min mixing on vortex. A plasma protein precipitant consisting of 0.5 mL of acetonitrile was then added. After that, the steps necessary to prepare the samples that would be utilized to generate the in-vitro calibration curves were carried out. Non-compartmental analysis (NCA) and the pharmacokinetic (PK) solver program were used to calculate the critical pharmacokinetic parameters^22^. The plasma concentration–time graphs were plotted, and numerous pharmacokinetic metrics were obtained afterward.

### Ethics approval and consent to participate

Since the research is involving human participants and human material, it has been performed in accordance with the Declaration of Helsinki and has been approved by an appropriate ethics committee. The named volunteers, who applied for approval for the study protocol from the ethical point of view, have confessed their complete awareness with information about the goals and risks of the volunteering methodology before being requested to sign a written consent form which was further approved by the institutional ethics committee. The Committee of Nahda University, Beni-Suef, Faculty of Pharmacy has approved the study protocol from an ethical point of view. Approval No: NUB-034-023. An informed consent was obtained from all subjects/participants.

## Results and discussion

### Optimization of the method

**Figure 2:**
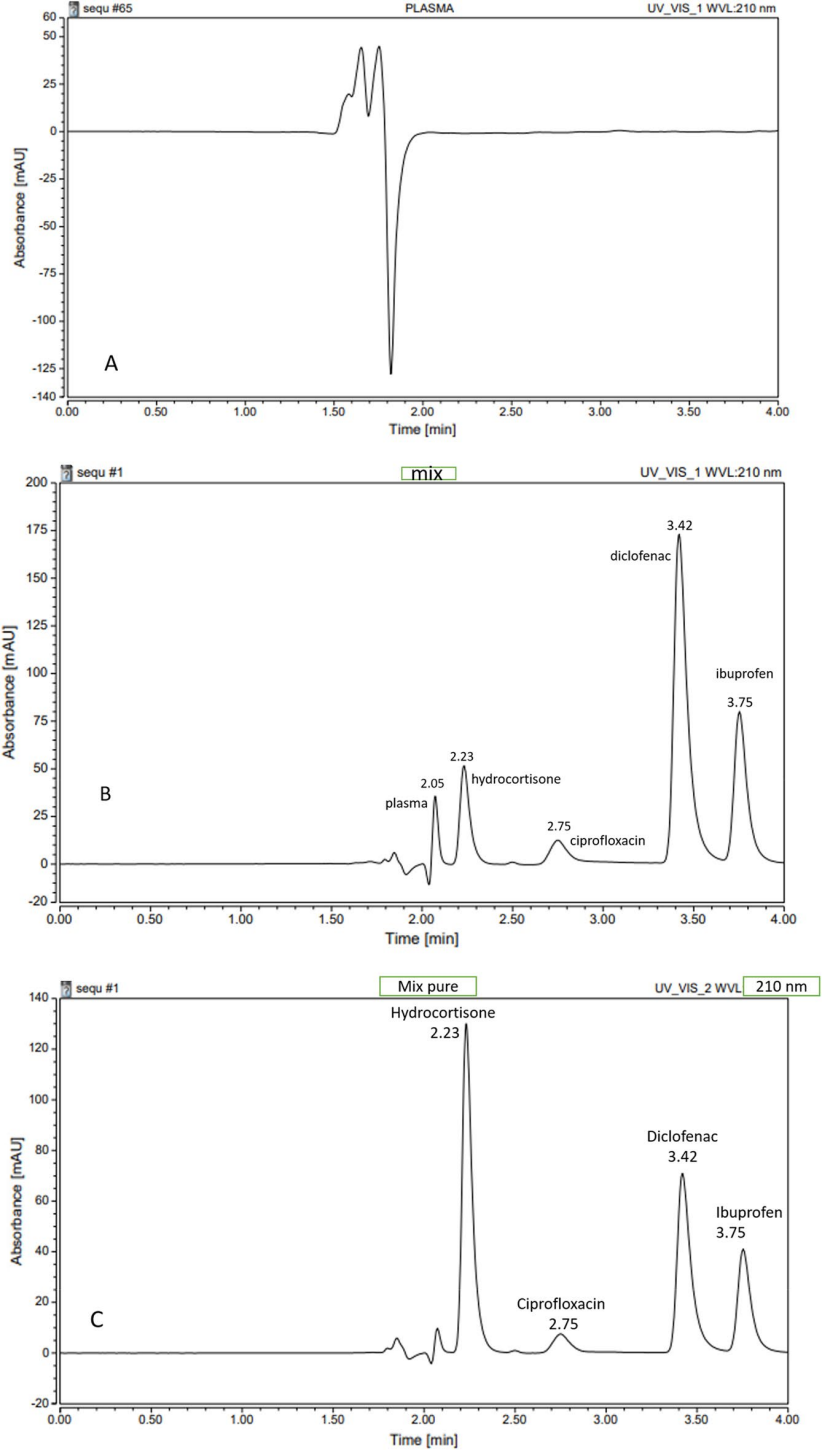
UPLC chromatogram of (**A**) pure plasma, (**B**) IS, and the studied medicines were added to a plasma sample, (**C**) a pure sample of the analyzed drugs and IS scanning at 210 nm.

A unique and fast method for the determination and separation of a mixture of three drugs viz*.,* ciprofloxacin (CIP), Ibuprofen (IBU), and diclofenac sodium (DIC) in actual samples of human plasma was developed. Separation was achieved within only 4-min analysis time (CIP 2.75 min), (DIC 3.42) and (IBU 3.75 min) Fig. 2. This method is special and gives sharp peaks in low retention time with high selectivity and sensitivity. The method's validation agreed with the requirements of the FDA, and the results were sufficient.

#### Choice of the appropriate solvent to precipitate proteins

It was attempted to precipitate proteins using many solvents. Ultimately, the protein was precipitated with acetonitrile since it decreases plasma component interference. After the complete dryness of the pure supernatant, the solution was refilled using a mixture of acetonitrile and water (pH 3 with diluted acetic acid). The latter solution was chromatographed using an X-bridge UPLC RP-C18 reversed-phase stationary phase column (2.1 × 150 mm, 1.7 μm). Literature consultation^23–25^ has proved the choice of acetonitrile as a perfect solvent for the separation of CIP, DIC, and IBU.

#### Mobile phase mixture and flow rate optimization

Several water and acetonitrile mobile phase mixes (from 60:40 to 35:65) and varying flow rates (from 0.1 to 0.2 mL/min) were tested to separate the analyzed drugs and the components of the plasma successfully, taking into consideration the necessity of a high concentration of acetonitrile to obtain a homogeneous, sharp peak with an adequate retention time for CIP, DIC, and IBU. It was observed that low pH was required to produce a perfect peak for CIP. Several pH values between 4 and 8 were explored to improve separation, but CIP was still connected with the plasma peak in every case. For entirely separate CIP and plasma, acetonitrile: water (pH 3 with diluted acetic acid) is used, along with the suitable separation time and sharpness of the peak for DIC and IBU. After applying many separation methods, a solution of 65% acetonitrile and 35% H_2_O (pH 3 by diluted acetic acid) and a flow rate of 0.2 mL/min for 4 min was selected. To raise the precision and accuracy of the results, internal standards are used to calculate the concentrations of additional compounds by calculating the outcome factor; however, they must not interfere with the sample's main components. Searching for various internal standards, hydrocortisone emerged as the one that performed success through creation and improvement. Procedure optimization followed with total separation Fig. 2.

### Validation of the method

The suggested technique was validated for the three drugs under test by US-FDA rules for industrial bioanalytical method validation to determine suitability, stability, LOD, LOQ, linearity, selectivity, accuracy, and precision (intraday and interday)^18^.

### Linearity

With various samples except zero, the method's linearity was evaluated (for pure drugs and spiked plasma standards). A linear regression analysis was utilized to assess the linearity, and calibration curves were used to measure the analyte concentrations. Plots were made against the standard concentrations using the peak area ratios of each medicine to the IS, which revealed the correctness of the computed correlation coefficients' values, slopes, and intercepts. The regression coefficients of the calibration curves applied for the calculation of tested analytes were of the value 0.99 and more, as illustrated in Table 2

**Table 2:** Parameters for the assay and technique validation for the suggested methods for determination of the studied medicines in pure & human plasma samples.

Parameters	Pure samples			Human plasma samples		
	CIP	DIC	IBU	CIP	DIC	IBU
Range (µg/mL)	0.5–20	3–100	3–100	0.3–10	1–25	0.2–11
Slope	1.6904	0.9898	0.4579	1.0074	1.0026	1.0037
Intercept	0.3067	0.0353	0.3629	0.0224	0.0342	0.0204
Correlation(r)	0.99	0.99	0.99	0.99	0.99	0.99

### The limit of detection (LOD) and quantitation (LOQ)

Based on the findings for three replicates, the LOD and LOQ of CIP, DIC, and IBU were calculated. CIP, DIC, and IBU had LOD values of 0.328, 0.98, and 0.39 µg/mL, respectively, corresponding to 0.439, 2.99, and 2.92 µg/mL LOQ values.

### Precision and accuracy

Furthermore, it was concluded that the lower limit of quantification is the fewest sample concentration that can be quantified by sufficient precision and accuracy (LLOQ). CIP, DIC, and IBU were all included in the LLOQ at concentrations of 3, 3, and 0.5 µg/mL, respectively. To evaluate intra-day and inter-day accuracy and precision at four different concentrations (LLOQ (low-quality control (LQC), moderate quality control (MQC), and high-quality control (HQC)), triplicates for each analyte were employed. As calculated by the coefficient of variation, precision was reported to be 0.65, 0.17, and 0.42% for CIP, DIC, and IBU for intraday precision, respectively, and 0.85, 0.35, and 0.81% for interday precision. (Table 3). Comparison of the LOD, LOQ, retention time, and precision of each of the studied drugs and other reported data^7,26^ was presented in Table 4.

**Table 3:** The proposed method's accuracy and precision (inter and intra-assay).

Component	Concentration (µg/mL)	Intraday		Interday	
		Recovery %	RSD%	Recovery %	RSD%
CIP	1	100.30	0.65	98.65	0.85
	4	99.10		97.40	
	10	100.09		99.06	
DIC	3	99.12	0.17	98.57	0.35
	9	99.15		99.01	
	25	99.43		99.25	
IBU	0.5	98.98	0.42	100.84	0.81
	2.5	98.85		99.24	
	11	98.63		99.72

**Table 4:** Comparison of the LOD, LOQ, retention time, and precision each of the studied drugs and other reported data.

Reported	LOQ (µg/L)	LOD (µg/L)	Retention time (min)	Precision	Type of samples
Ciprofloxacin	0.439	0.328919	2.75	0.65	Human plasma samples (This study )
Diclofenac	2.9945	0.9882	3.42	0.17	
Ibuprofen	2.92	0.39	3.75	0.42	
Reported 1^7^	5	0.1	6.1	–	Wastewater
	120	12	5.5	–	
	200	20	9.7	–	
Reported 2^26^			16	1.03	Wastewater
	0.058–0.752	0.019–0.247	7.9	1.28	
			7.4	0.62	

This study is unique and perfect from others due to using real human plasma samples.

Reports 1 and 2 use wastewater samples. There are clear improvements in the method proved.

by the obtained low values of retention time, LOQ, and LOD compared to other reports.

### Extraction recovery

Peak areas of the samples loaded into pure plasma at the QC levels (LQC, MQC, and HQC) were compared with peak areas of the standard analytes to determine the extraction recovery of the studied drug using the proposed methods. In Table 5, the obtained extraction recoveries (%RSD 6%) verified the extraction technique's accuracy and stability.

**Table 5:** Recovery results of the medications under research spiked with human plasma.

Drug	Concentration of the drug (µg/mL)	% Recovery
CIP	1	91.67
	4	101.81
	10	100.68
Mean ± %RSD		98.05 ± 5.67
DIC	3	95.05
	9	99.30
	25	95.86
Mean ± %RSD		96.74 ± 2.33
IBU	0.5	93.99
	2.5	95.39
	11	99.38
Mean ± %RSD		96.26 ± 2.90
IS	5	88.58 ± 0.11

### Selectivity

To test selectivity, the spectral data of plasma samples loaded with IS and each tested drug was compared with the ones from plasma samples of fit volunteers. It was tested using the separation among the tested drugs, internal standard, and plasma particles, as in Fig. 2.

### Stability

Stability tests have been done to examine the stability of the drugs in plasma samples under a wide range of conditions that can arise during sample analysis. Three freeze cycles, as well as the bench top stability, were finished. All the results in Table 6 demonstrate the stability of the samples. Standard CIP, DIC, and IBU solutions stood stable for one week when frozen.

**Table 6:** Stability of the studied drugs spiked with human plasma at various conditions.

Drugs	The concentration of the drug (µg/mL)	Three freeze-dissolve periods	Benchtop stability
		% Recovery ± %RSD	% Recovery ± %RSD
CIP	1(LQC)	100.00 ± 0.57	91.30 ± 0.69
	4(MQC)	99.20 ± 0.15	102.00 ± 1.42
	10(HQC)	100.00 ± 0.52	90. 60 ± 0.16
DIC	3(LQC)	96.04 ± 0.34	83.58 ± 0.99
	9(MQC)	97.42 ± 0.11	101.36 ± 0.16
	25(HQC)	98.58 ± 0.06	95.32 ± 0.02
IBU	0.5(LQC)	99.35 ± 0.19	93.92 ± 0.53
	2.5(MQC)	102.15 ± 1.16	95.92 ± 0.63
	11(HQC)	100.41 ± 0.13	99.23 ± 0.13

### Greenness profile of the created method

**Figure 3:**
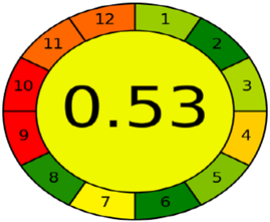
The score of the green metric AGRee for the new method.

The idea of "green analytical chemistry" (GAC) enables analytical chemists to consider safety, health, and environmental issues while doing the work. A technique is considered a superior green analysis if the Eco-scale score exceeds 75. The procedure will be more environmentally friendly as the score becomes close to 100. The new UPLC method could be seen as a perfect green method because it pointed to a score of 84. The Eco-scale score is shown in Table 7. AGREE is a green tool can be considered a rapid quantitative technique that provides a score indicating how closely a technique agrees with the 12 essential rules of green analytical chemistry. Higher scores indicate greener approaches; the total score is shown in the circle pictogram's center Fig. 3.

**Table 7:** The Penalty points (PPs) of the proposed method per the analytical Eco-Scale.

	Analytical Eco-scale	PPs
Reagents	Acetonitrile	4
	Acetic acid	4
Instruments	UPLC ≤ 1.5 KWh/sample	1
	Centrifuge	1
	Ultrasonic	1
	Waste	5
	Total pp	16
	Eco–scale	84

### Results of a pharmacokinetic study

**Figure 4:**
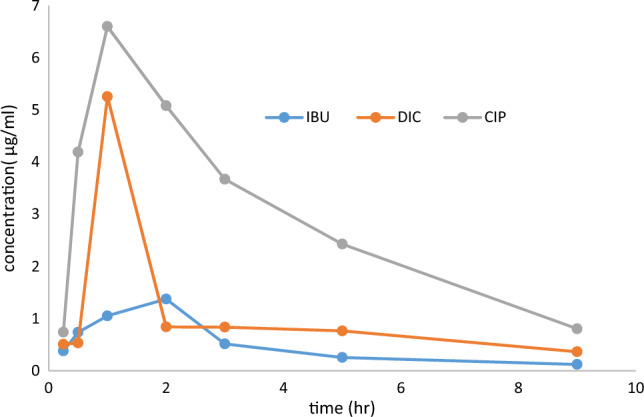
Mean plasma concentration–time curves of CIP after intake of CIP tablet 750 mg orally, DIC after intake of Diclac 75 ID tablet 75 mg orally, and IBU after intake of Brufen 600 mg tablet orally.

Table 8 displays the pharmacokinetic parameters determined using non-compartmental analysis and calculated after the oral administration of CIP, DIC, and IBU. It investigated how to measure the given drugs' pharmacokinetic properties individually. Also, the examined drugs' mean plasma concentration–time profiles (n = 3) are displayed in Fig. 4. CIP and DIC took 1 h, and IBU took 2 h to achieve their maximum plasma concentrations (T_max_). The maximum plasma concentrations (C_max_) for CIP^27–29^, DIC^30,31^, and IBU^32,33^ were 6.6, 5.25, and 1.37 µg/mL, respectively.

**Table 8:** Pharmacokinetic study results of the proposed HPLC technique.

Parameter	Unit	CIP (750 mg)	DIC (75mg)	IBU (600mg)
Absorption parameters				
T_max_	h	1	1	2
C_max_	µg/mL	6.6	5.256	1.37
AUC_0-t_	µg/mL*h	26.88	8.277	4.31
AUC_0-inf_	µg/mL*h	30.22	10.783	4.83
Elimination parameters				
T_1/2_	h	2.87	4.77	2.96
Mean residence time (MRT)	h	4.46	6.14	3.91
Clearance (Cl/F)	L/h	27.29	76.506	124.09
Distribution parameters				
Volume of distribution (Vz/F)	L	113.28	526.68	531.39

## Conclusion

A quick procedure for determining and separating CIP, IBU, and DIC in real human plasma samples has been developed. Hydrocortisone was chosen as the internal standard (IS). The drugs were separated chromatographically using an Acquity UPLC ® BEH C18 1.7µm (2.1 X150 mm) column applying (UPLC) with UV detection at 210 nm. Calibration curves were discovered to be linear with acceptable correlation coefficients (0.99%). Exceptional precision and accuracy, limit of detection (LOD) and quantitation (LOQ), selectivity, and stability have been shown. Separation was achieved in only a 4-min analysis time. Two green metrics were applied, the Analytical Eco-scale and the Analytical GREEnness Calculator (AGREE). The investigation's focus was increased to investigate each of the three drugs' pharmacokinetic characteristics. FDA guidelines were applied in the method's validation, and the results were satisfactory. The new approach is more effective regarding its impact on the natural world and people's health, as well as the budget of analysis and sample preparation, according to comparisons made with the method employing the greenness metrics.

## Data Availability

This paper contains all data and analyses created or analyzed during the current investigation.
